# Sunstroke, or Thermic Fever

**Published:** 1885-09

**Authors:** 


### Sunstroke, or Thermic Fever.

No error can be fraught with more dangerous consequences than that of failing to discriminate between heat exhaustion and true sunstroke. The former is comparatively a mild affection, which does not differ in symptoms from any other form of acute exhaustion. It is characterized by dilated pupils, a cold, pale and perspiring skin, a quick but feble pulse, with great general prostration, and a tendency to syncope. Recovery ensues within twenty-four hours under rest and the administration of stimulants.

True sunstroke, or *coup de soleil* is a far more terrible affection. It is characterized by contracted pupils, a hot, dry and flushed skin, rapid and forcible pulse, throbbing carotids, labored or stertorous breathing, with profound coma, or delirium and convulsions ending in coma. In the fulminant cases that have been observed, the unfortunate persons have dropped dead as if struck a mortal blow by an unseen hand. Contrary to the popular opinion, it is not necessary that the patient should have been exposed to the direct rays of the sun. For, as was noticed by many distinguished observers, and practically demonstrated by Dr. H. C. Wood, Jr., in his experiments on animals, excessive heat, heat alone, is the essential factor in this disease. Many of the worst cases have occurred at night, in houses, ih tents, and in narrow defiles, where the sun never entered, but where the atmosphere was hot and stifling. It is, therefore, a true fever, and, as suggested by Dr. Wood, should be designated thermic fever, as expressive of its exciting cause.

The treatment, which must be instituted promptly, can be summed up in three words—reduce the temperature. It is the extraordinary high temperature which is burning up the patient, and which, unless speedily reduced, will cause death by paralysis of the heart. He should, therefore, be at once removed to a shady place in the fresh air, his head slightly elevated, and his whole body, especially his head and chest, kept deluged with ice water. An ice cap, in addition, should be applied to the back of the head, until his temperature and pulse have fallen. Aconite internally will also probably be found beneficial in controlling the circulation. Morphia, hypodermatically, has been foun i to be of great value in cases characterized by restlessness and convulsions. If the attack has come on shortly after a meal, there can be no doubt of the propriety of at once unloading the stomach by an emetic. If the patient is insensible, apomorphia, gr. one tenth, may be given hypodermatically. The Australian physicians produce emesis in these cases by the rectal injection of twenty grains of ipecac. They have always noticed an abatement of the symptoms as soon as vomiting began.—*Medical Bulletin*

